# Arabidopsis Cys2/His2 Zinc Finger Transcription Factor ZAT18 Modulates the Plant Growth-Defense Tradeoff

**DOI:** 10.3390/ijms232315436

**Published:** 2022-12-06

**Authors:** Weiwei Li, Min Zhang, Tingyu Zhang, Yueyan Liu, Lijing Liu

**Affiliations:** The Key Laboratory of Plant Development and Environmental Adaptation Biology, Ministry of Education, School of Life Sciences, Shandong University, Qingdao 266237, China

**Keywords:** the growth-defense tradeoff, hormone signaling, ZAT18, transcription factor, salicylic acid, auxin

## Abstract

Plant defense responses under unfavorable conditions are often associated with reduced growth. However, the mechanisms underlying the growth-defense tradeoff remain to be fully elucidated, especially at the transcriptional level. Here, we revealed a Cys2/His2-type zinc finger transcription factor, namely, ZAT18, which played dual roles in plant immunity and growth by oppositely regulating the signaling of defense- and growth-related hormones. ZAT18 was first identified as a salicylic acid (SA)-inducible gene and was required for plant responses to SA in this study. In addition, we observed that ZAT18 enhanced the plant immunity with growth penalties that may have been achieved by activating SA signaling and repressing auxin signaling. Further transcriptome analysis of the *zat18* mutant showed that the biological pathways of defense-related hormones, including SA, ethylene and abscisic acid, were repressed and that the biological pathways of auxin and cytokinin, which are growth-related hormones, were activated by abolishing the function of *ZAT18*. The ZAT18-mediated regulation of hormone signaling was further confirmed using qRT-PCR. Our results explored a mechanism by which plants handle defense and growth at the transcriptional level under stress conditions.

## 1. Introduction

As sessile organisms, plants must struggle against various pathogens and herbivores, including bacteria, fungi, viruses and insects. To fight these invasions, plants activate defense responses, including pattern-triggered immunity (PTI) and effector-triggered immunity (ETI), to protect themselves [1,2]. However, constitutive activation of defense responses is an energy-consuming process and generally results in growth penalties [3,4,5,6]. For example, prolonged treatment with pathogen-associated molecular patterns (PAMPs), which activates PTI responses, significantly inhibits Arabidopsis growth [7]. This phenomenon is called the growth-defense tradeoff. Understanding the mechanisms underlying the growth-defense tradeoff is critical for breeding disease-tolerant crops. A good example is the study of TL1-binding transcription factor 1 (TBF1). The translation of TBF1 is controlled by upstream open reading frames (uORFs), which allows TBF1 to only be translated to activate immune responses and repress growth upon pathogen infection [8]. The uORFs of TBF1 were later used as a key regulatory element to balance rice immunity and growth in molecular breeding [9].

Numerous works verified that hormone crosstalk plays fundamental roles in regulating the growth-defense tradeoff [10,11,12]. Among all hormones, the accumulation of salicylic acid (SA) is observed during both PTI and ETI and plays a key role in plant defenses against biotrophic and hemibiotrophic pathogens [13]. Upon pathogen infection, SA is synthesized through the ICS pathway [14,15]. Then, the accumulated SA is detected by NPR proteins, inducing the expression of SA-responsive genes through TGA transcription factors [16,17]. Moreover, SA is also involved in plant growth inhibition [18,19]. For example, SA binds to and inhibits the activity of the A subunit of protein phosphatase 2A, which upregulates the signaling of the growth-related hormone auxin by modulating the phosphorylation status of auxin transporter PIN2 [20] to dampen growth under pathogen infection conditions. SA can also inhibit plant growth by modulating gibberellic acid (GA) signaling. Yu et al. found that the SA receptor NPR1 mediates the ubiquitination and degradation of the GA receptor GID1 to suppress GA signaling [21]. Moreover, SA was reported to regulate the expression of auxin signaling at the transcriptional level [22]. However, the transcription factors that mediate SA-auxin crosstalk are poorly understood.

The zinc finger of *Arabidopsis thaliana* (ZAT) proteins belong to the C1-2i subclass of C2H2-type zinc finger proteins [23]. Except for the zinc finger domain, the majority of this subclass contains conserved a DNA binding motif (QALGGH), nuclear localization signal at the N-terminus and ethylene-responsive element-binding factor-associated amphiphilic repression (EAR) motif, which generally represses transcription in plants at the C-terminus [23]. Many ZAT proteins are involved in the regulation of the plant growth and defense tradeoff under abiotic conditions. For example, the expression of *ZAT10* is strongly induced by abscisic acid (ABA) and multiple abiotic stresses, including salt, drought and cold [24,25]. Overexpression of *ZAT10* enhances plant resistance to abiotic stresses, which is accompanied by growth inhibition [24,25,26,27]. Even though ZAT10 has an EAR motif, it has a dual role in the transcription of cadmium stress-related genes and acts as a positive transcription regulator to increase resistance to osmotic stress [28,29]. Another study also showed that *ZAT7* overexpression enhances plant tolerance to salinity stress with a growth penalty [30]. To date, the mechanisms by which ZATs contribute to growth inhibition after pathogen infection remain elusive.

In this study, we first identified Arabidopsis Cys2/His2 zinc finger transcription factor *ZAT18* as a SA-responsive gene. Abolishing the function of ZAT18 compromised plant responses to SA and pathogens. *ZAT18* overexpression enhanced plant defense but was accompanied by growth inhibition and auxin-deficient phenotypes. By comparing the transcriptome of the WT and *zat18* mutant, we discovered that ZAT18 was a key factor in activating genes involved in defense responses, including the SA, ethylene (ET) and abscisic acid (ABA) pathways, and repressing genes regulating growth, especially the auxin and cytokinin (CK) pathways. Previously, Yin et al. reported that ZAT18 was induced by drought and enhanced plant drought tolerance [31]; therefore, we concluded that ZAT18 is activated to oppositely regulate plant defense and growth signaling under both biotic and abiotic stress conditions.

## 2. Results

### 2.1. ZAT18 Mediated Plant Responses to SA

**Figure 1:**
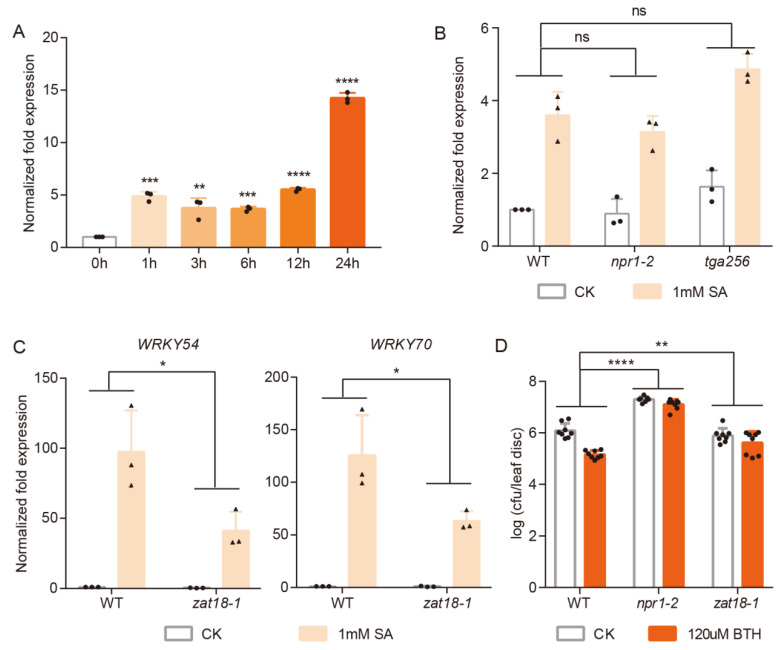
*ZAT18* is an SA-induced gene that encodes a protein that is important for SA responses. (**A**) Two-week-old wild-type (WT) seedlings were dipped with 1 mM SA, and samples were collected at the indicated time points to examine the expression levels of *ZAT18* with *UBQ5* as a reference. Significant differences were detected using Student’s *t*-test. Data are shown as mean ± s.d. (n = 3, n indicates the number of biological replicates). (**B**) The expression levels of *ZAT18* in the WT and *npr1-2* and *tga2/5/6* mutants after sterile dH_2_O (CK) or 1 mM SA treatment for 1 h. Experiments were performed as described in (**A**). Significant differences were detected using two-way ANOVA. Data are shown as mean ± s.d. (n = 3, n indicates the number of biological replicates). (**C**) The expression levels of *WRKY54* and *WRKY70* in the WT and *zat18-1* mutant after sterile dH_2_O (CK) or 1 mM SA treatment for 1 h. Experiments were performed as described in (**A**). n = 3, n indicates the number of biological replicates. (**D**) Three-week-old WT and *zat18-1* and *npr1-2* mutants were pre-treated with dH_2_O (CK) or 120 μM BTH for 1 day before infiltration with *Psm* ES4326 (OD_600nm_ = 0.001). The bacterial titer was checked at 3 days post-inoculation (dpi). cfu, colony forming unit. n = 8, n indicates the number of biological replicates. * *p* < 0.05; ** *p* < 0.01; *** *p* < 0.001; **** *p* < 0.0001; ns, no significant difference.

To further study SA signaling, we performed transcriptome analysis on samples that were treated with SA for 1 h. Three biological samples were collected for both CK and SA treatment and we observed that *ZAT18* was significantly induced in two SA-treated samples (Figure S1). The SA-induced expression pattern of *ZAT18* was further confirmed using qRT-PCR. We found that SA upregulated the expression of *ZAT18* at all time points we checked (1 to 24 h after the SA treatment) (Figure 1A). As NPR1 is the SA receptor that controls the expression of most SA-responsive genes and TGA2/5/6 are transcription factors that work downstream of NPR1 [32,33], we examined whether SA activates *ZAT18* in an NPR1- and TGA-dependent manner. As shown in Figure 1B, the SA-induced expression of *ZAT18* showed no significant difference between the WT and *npr1-2* or *tga2/5/6* mutants, indicating that the SA induction of *ZAT18* was independent of NPR1 and TGA2/5/6.

Then, we wondered whether ZAT18 could mediate the plant responses to SA. For this purpose, a T-DNA insertion *ZAT18* knockout mutant was identified (Figure S2). Two-week-old seedlings of the WT and *zat18* mutant (*zat18-1*) were treated with 1 mM SA for 1 h to measure the expression of the SA-responsive genes, namely, *WRKY54* and *WRKY70*. We found that the *ZAT18* mutation significantly dampened the SA-induced expression of these genes (Figure 1C), indicating that ZAT18 was required for the plants to fully respond to SA. Next, we tested whether ZAT18 modulates SA-triggered plant resistance to pathogens. For this purpose, three-week-old WT and *zat18-1* were pretreated with 120 μM benzothiadiazole (BTH), which is a chemical analog of SA and induces plant defense that is dependent on SA signaling [33]. One day later, the plants were further infiltrated with *Pseudomonas syringae* pv.*maculicola* (*Psm*) ES4326. The pathogen levels in the WT and *zat18-1* were measured at three days after the infection. We observed that pretreatment with BTH enhanced the plant resistance to *Psm* ES4326 in the WT, where the bacteria titer reduced from 10^6.02^ to 10^5.04^, but not in the *npr1-2* mutant as expected. However, the BTH-triggered resistance was also significantly compromised in the *zat18-1* mutant (Figure 1D).

**Figure 2:**
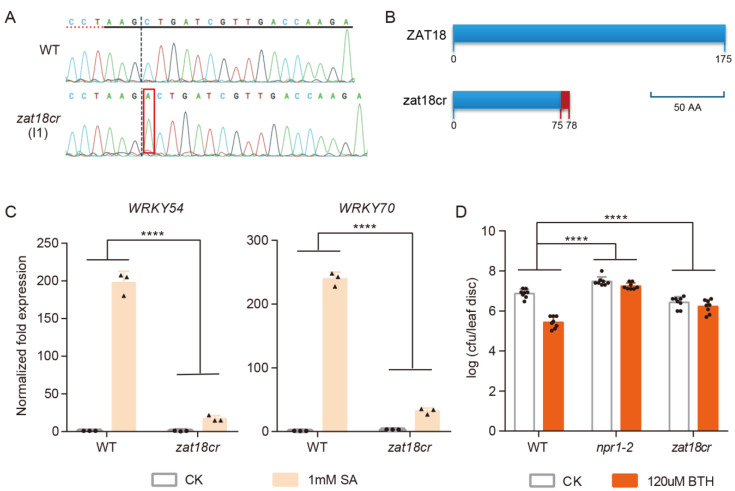
Generation of the *zat18cr* mutant and its SA-related phenotypes. (**A**) The Sanger sequencing chromatograms of WT and *zat18cr* are shown and the inserted A is marked. (**B**) The protein length encoded by *ZAT18* in the WT and *zat18cr*. The light blue bar indicates the WT sequence of ZAT18 and the red bar represents the unmatched sequence. The bar graph of 50 AA represents the length of 50 amino acids (AAs) in the ZAT18 and zat18cr structures. (**C**) The expression levels of *WRKY54* and *WRKY70* in the WT and *zat18cr* mutant after sterile dH_2_O (CK) or 1 mM SA treatment for 1 h. Experiments were performed as described in Figure 1A. n = 3, n indicates the number of biological replicates. (**D**) BTH-induced resistance in the WT and *zat18cr* and *npr1-2* mutants. Experiments were performed as described in Figure 1D. n = 8, n indicates the number of biological replicates.**** *p* < 0.0001.

To confirm the role of ZAT18 in SA signaling, we further mutated *ZAT18* using the Clustered Regularly Interspaced Short Palindromic Repeats (CRISPR)-associated 9 (CRISPR/Cas9) genome editing tool [34]. A 20 nt specific spacer against the first exon of *ZAT18* with the AGG protospacer-adjacent motif (PAM) sequence was chosen using the CRISPR program (http://crispor.tefor.net/, accessed on 13 December 2020) (Figure S2A). Successfully edited *zat18* mutants were identified in the T_1_ generation. A homozygous line with an A insertion that resulted in early termination of ZAT18 translation was chosen for further experiments and was termed *zat18cr* (Figure 2A,B). We observed that the SA induction of *WRKY54* and *WRKY70* was dampened in *zat18cr* (Figure 2C). Furthermore, BTH-triggered resistance to *Psm* ES4326 was also abolished in this mutant (Figure 2D). All these results indicated that ZAT18 played an important role in the SA signaling.

### 2.2. ZAT18 Enhanced Plant Resistance to Psm ES4326

**Figure 3:**
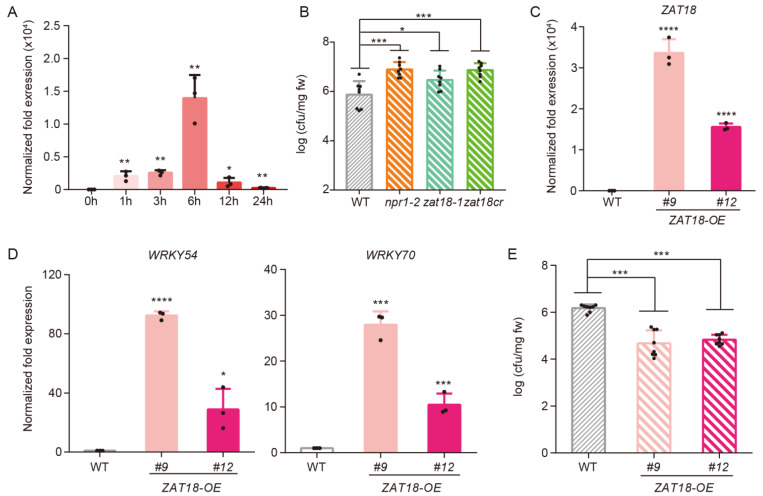
ZAT18 enhances the plant defense against *Psm* ES4326. (**A**) Three-week-old wild-type (WT) seedlings were infiltrated with *Psm* ES4326 (OD_600nm_ = 0.001) and samples were collected at indicated time points to examine the expression levels of *ZAT18* with *UBQ5* as a reference. n = 3, n indicates the number of biological replicates. (**B**) Three-week-old WT and *npr1-2* and *zat18* mutants were sprayed with *Psm* ES4326 (OD_600nm_ = 0.01) and the third to sixth true leaves were collected to measure the bacterial titer at 3 dpi. cfu, colony forming unit. n = 8, n indicates the number of biological replicates. (**C**,**D**) The expression levels of *ZAT18* (**C**) and *WRKY54* and *WRKY70* (**D**) in three-week-old WT and *ZAT18-OE* seedlings without any treatment. n = 3, n indicates the number of biological replicates. (**E**) Three-week-old WT and *ZAT18-OE* lines were sprayed with *Psm* ES4326 (OD_600nm_ = 0.1) and the third to sixth true leaves were collected to measure the bacterial titer at 3 dpi. cfu, colony forming unit. n = 8, n indicates the number of biological replicates. * *p* < 0.05; ** *p* < 0.01; *** *p* < 0.001; **** *p* < 0.0001.

SA signaling mediates plant defense against biotrophic and hemibiotrophic pathogens, such as *Psm* ES4326 [35]. As ZAT18 regulates SA signaling, we hypothesized that ZAT18 also manages plant basal defense against *Psm* ES4326. To test this speculation, the expression of *ZAT18* after *Psm* ES4326 inoculation was first examined. Compared with untreated samples, a 2000-fold induction of *ZAT18* was observed at 1 h post-infection (hpi) and the expression peak showed a 14,000-fold induction at 6 hpi (Figure 3A). Then, to examine whether the *ZAT18* mutation dampens plant defense against *Psm* ES4326, a higher dosage of *Psm* ES4326 (OD_600nm_ = 0.01) was sprayed onto the three-week-old WT and *zat18* mutants. As shown in Figure 3B, a higher bacterial titer was detected in the *zat18-1* and *zat18cr* mutants than in the WT, indicating that ZAT18 was required for the plant basal defense against *Psm* ES4326.

To further confirm that ZAT18 positively regulates plant basal defense, we made a construct to express *ZAT18* driven by the *35S* promoter and transformed it into the WT. Two independent *ZAT18* overexpression (*ZAT18-OE*) lines (*ZAT18-OE #9* and *ZAT18-OE #12*), with 3.3 × 10^4^-fold and 1.6 × 10^4^-fold expressions of *ZAT18* compared with that in the WT, were identified and used in the following experiments (Figure 3C). As ZAT18 positively regulates SA signaling, the expression of SA-responsive genes was tested in the *ZAT18-OE* lines. Consistent with expectations, the expression of *WRKY54* and *WRKY70* was upregulated in the *ZAT18-OE* lines (Figure 3D). Moreover, the expression of *PR* genes, which are SA late responsive genes, was also upregulated in the *ZAT18-OE* lines (Figure S3). Then, three-week-old *ZAT18-OE* lines were inoculated with *Psm* ES4326 to determine whether *ZAT18* overexpression could enhance the plant basal resistance to pathogens. As shown in Figure 3E, *ZAT18* overexpression reduced the pathogen titer from 10^6.17^ to 10^4.66^ and 10^4.82^, respectively, in the two transgenic lines. These data further confirmed that ZAT18 positively regulated the plant basal defense responses.

### 2.3. ZAT18 Suppressed Plant Growth by Repressing Auxin Signaling

**Figure 4:**
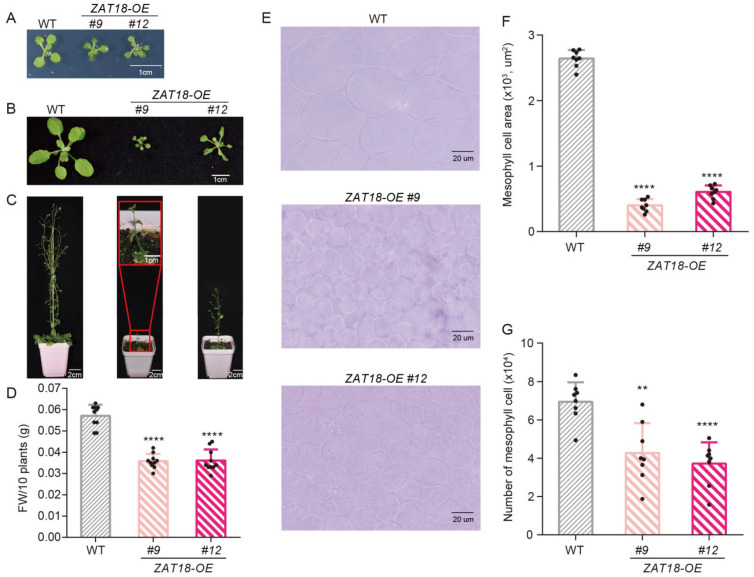
*ZAT18* overexpression resulted in growth inhibition. (**A**–**C**) Representative plants of ten-day-old (**A**), three-week-old (**B**) and eight-week-old (**C**) WT and *ZAT18-OE* lines. (**D**) Ten-day-old WT and *ZAT18-OE* seedlings were collected and weighed. n = 10, n indicates the number of biological replicates. (**E**) Mesophyll cells of the WT and *ZAT18-OE* lines were observed from a paradermal view. The fifth and sixth true leaves from the three-week-old plants were collected, fixed and cleared for microscopic analysis. Bar: 20 μm. (**F**) The areas of mesophyll cells in (**E**) were measured using ImageJ. n = 8, n indicates the number of biological replicates. (**G**) The numbers of mesophyll cells per leaf were determined via dividing the leaf size by the average cell area of the corresponding leaf. n = 8, n indicates the number of biological replicates. ** *p* < 0.01; **** *p* < 0.0001.

During our experiments, we observed that *ZAT18-OE* lines looked smaller than the WT. To determine the role of ZAT18 in plant growth, we carefully observed the morphological phenotypes of ten-day-old, three-week-old and eight-week-old plants of the WT, *zat18* mutants and *ZAT18-OE* lines. No significant differences were observed between the WT and *zat18* mutants at these stages (Figure S4). At all these stages, the *ZAT18-OE* lines were smaller than the WT (Figure 4A–D). *ZAT18* overexpression significantly decreased the leaf cell size and cell number in each leaf (Figure 4E–G). The mean cell size was 2642 μm^2^ for the WT leaves and there were an average of 6.9 × 10^4^ cells in each WT leaf, while in the *ZAT18-OE* lines, the mean cell sizes were reduced to 400 μm^2^ and 604 μm^2^ and there were only 4.3 × 10^4^ and 3.7 × 10^4^ cells per leaf, respectively. These results indicated that ZAT18 may regulate cell division and expansion during development.

**Figure 5:**
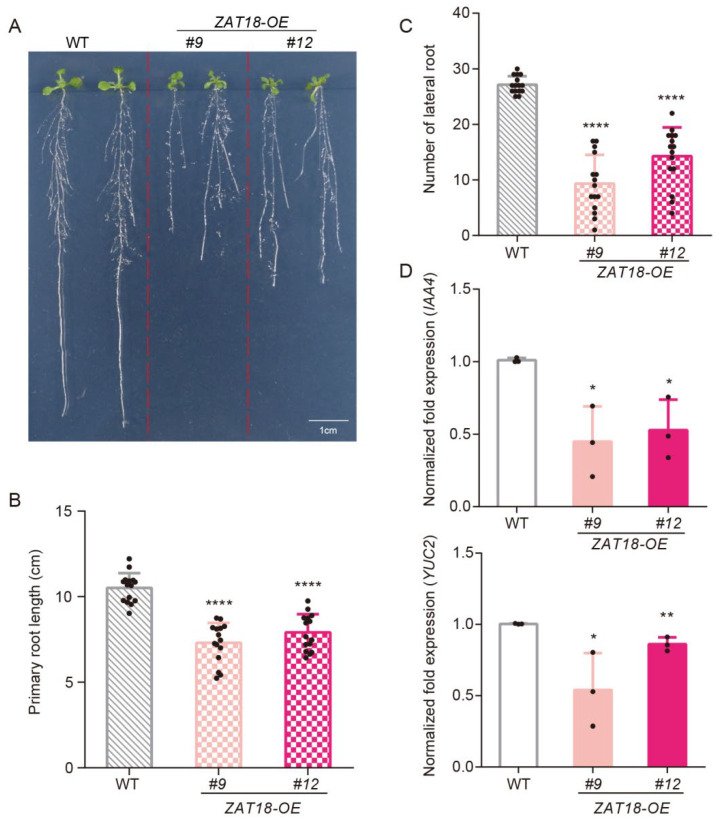
*ZAT18* overexpression lines showed auxin-deficient phenotypes. (**A**) Representative seedlings of two-week-old WT and *ZAT18-OE* lines to show the growth status of their roots. (**B**) The primary root lengths of the two-week-old WT and *ZAT18-OE* lines were measured using ImageJ. n = 15, n indicates the number of biological replicates. (**C**) The numbers of visible lateral roots of the two-week-old WT and *ZAT18-OE* lines. n = 15, n indicates the number of biological replicates. (**D**) The expression levels of *IAA4* and *YUC2* in the two-week-old WT and *ZAT18-OE* seedlings without any treatment. n = 3, n indicates the number of biological replicates. * *p* < 0.05; ** *p* < 0.01; **** *p* < 0.0001.

Meanwhile, *ZAT18-OE* lines produced short primary roots and fewer lateral roots (Figure 5A). Compared with the WT, the primary root length of the two-week-old seedlings was reduced by approximately 3 cm and the number of lateral roots was reduced from 27 to 9 and 14, respectively, in the two *ZAT18-OE* lines (Figure 5B,C). All these phenotypes of *ZAT18-OE* lines are reminiscent of auxin-insensitive or auxin-deficient mutants, indicating that ZAT18 may regulate auxin signaling [36,37,38]. Thus, we further checked the expression levels of the auxin synthesis gene *YUC2* and auxin-responsive gene *IAA4* in the *ZAT18-OE* lines. Consistent with their phenotypes, the expression levels of these genes were significantly reduced when *ZAT18* was overexpressed in plants (Figure 5D). To exclude the possibility that the *ZAT18-OE* lines mimicked auxin-deficient mutants due to elevated SA signaling, we further checked whether auxin signaling is affected in the *zat18* mutant. Even though the *zat18* mutant showed no morphological differences from the WT plants, the expression of auxin-related genes was upregulated in the *zat18* mutants (Figure S5), confirming the repression of auxin signaling by ZAT18.

### 2.4. ZAT18 Oppositely Regulated Defense- and Growth-Related Pathways

**Figure 6:**
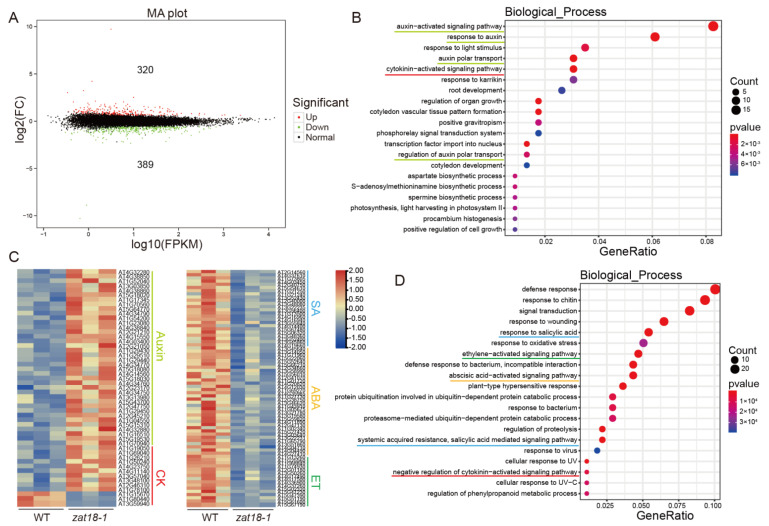
Transcriptome changes in the *zat18-1* mutant. Dozens of three-week-old WT and *zat18-1* mutant leaves were collected and RNA was extracted for sequencing. (**A**) The number of DEGs (differently expressed genes) up/downregulated in the *zat18-1* mutant. (**B**) Gene Ontology (GO) term enrichment (*p* < 0.05) of the genes that were specifically upregulated in the *zat18-1* mutant. (**C**) The relative expression levels of DEGs involved in hormone pathways. (**D**) GO term enrichment (*p* < 0.05) of the genes that were specifically downregulated in the *zat18-1* mutant. Hormone-related pathways are indicated by lines with different colors.

Because ZAT18 is a Cys2/His2-type transcription factor [31], we further performed transcriptome analysis to deeply understand its function in the growth-defense tradeoff. Two-week-old WT and *zat18-1* seedlings were collected and three biological replicates for each genotype were sent out for sequencing. The sequencing metrics of all RNA-seq libraries are shown in Table S1. Compared with the WT, 320 differently expressed genes (DEGs) were upregulated and 389 DEGs were downregulated in the *zat18-1* mutant (Figure 6A). We further analyzed which biological processes were regulated by ZAT18. As shown in Figure 6B,C, the auxin-related pathway was significantly enriched in the 320 upregulated DEGs, as presented by genes involved in the auxin-activated pathway, response to auxin, auxin polar transport and regulation of auxin polar transport. Moreover, the cytokinin-activated signaling pathway and negative regulation of cytokinin-activated signaling pathway was enriched in the upregulated DEGs and downregulated DEGs, respectively, indicating that ZAT18 suppressed cytokinin (CK) signaling (Figure 6B–D). Other biological pathways, including regulation of organ growth, photosynthesis, light harvesting in photosystem II, positive regulation of cell growth, and root and cotyledon development, which are related to plant growth, were also enriched in the upregulated genes (Figure 6B). These results indicated that the regulation of ZAT18 on plant growth is a complex process.

SA signaling was clearly downregulated in the *zat18-1* mutant because we observed enrichment in biological processes such as the response to salicylic acid, systemic acquired resistance and salicylic acid-mediated signaling pathways (Figure 6C,D). These results further confirmed that ZAT18 was a positive regulator of SA signaling. Except for SA, the genes related to biotic and abiotic defense were enriched, which was represented by the response to chitin, defense response to bacterium, incompatible interaction, defense response, cellular response to UV and response to oxidative stress. The downregulation of abiotic stress-related genes was consistent with previous data showing that ZAT18 is induced by abiotic stresses and enhances plant resistance to drought [31]. The regulation of biotic and abiotic stresses by ZAT18 may be achieved through phytohormones because in addition to SA signaling, the signaling of ABA and ethylene (ET), which are two other defense-related hormones, was also downregulated in the *zat18-1* mutant (Figure 6C,D).

**Figure 7:**
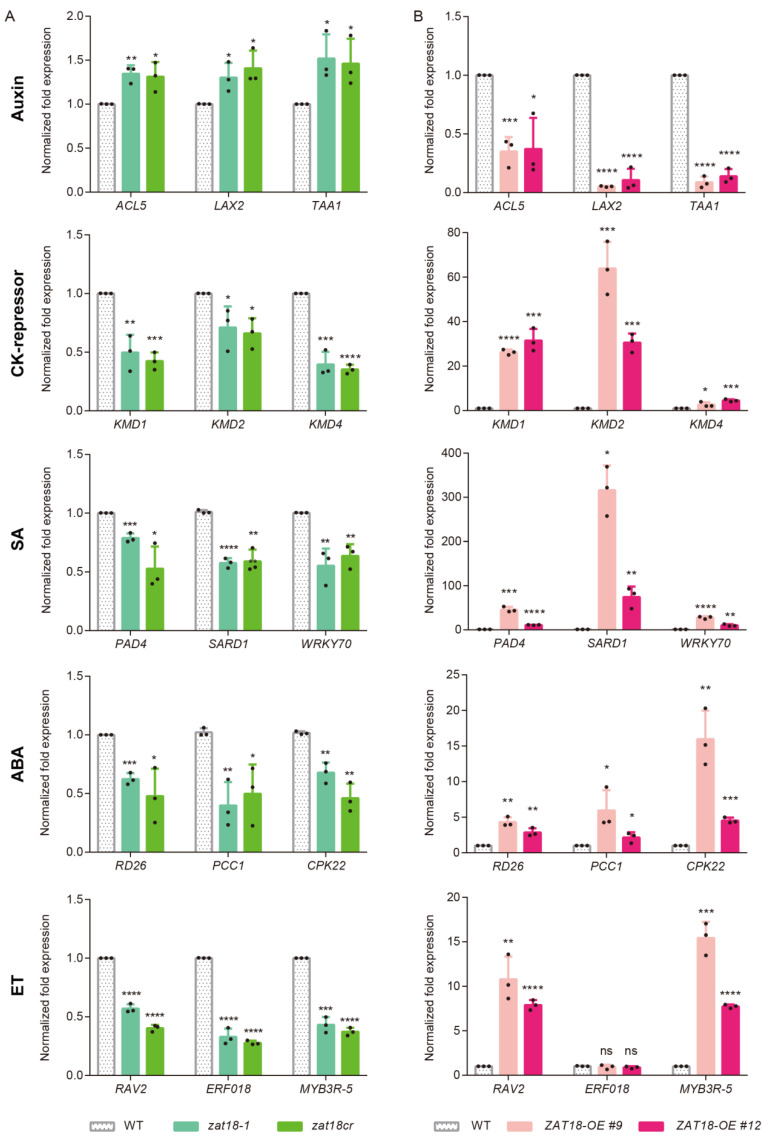
Validation of the expression levels of hormone-related DEGs in the *zat18* mutant and *ZAT18-OE* lines using qRT-PCR. (**A**) The expression levels of representative hormone-related genes identified using RNA sequencing of the *zat18* mutants. (**B**) The expression levels of representative hormone-related genes identified using RNA sequencing of the *ZAT18-OE* lines. n = 3, n indicates the number of biological replicates. * *p* < 0.05; ** *p* < 0.01; *** *p* < 0.001; **** *p* < 0.0001; ns, no significant difference.

To verify the RNA-seq results, qRT-PCR was performed to confirm the expression of these hormone-related genes in two *zat18* mutants. Three genes from each hormone pathway were selected. As shown in Figure 7A, the genes related to SA, ABA and ET signaling were downregulated, while genes in the auxin pathways were upregulated in both *zat18* mutants. As CK-activated genes and the negative regulators of CK response were enriched in upregulated and downregulated DEGs, respectively, qRT-PCR was performed on both categories. The CK activated genes were slightly upregulated (Figure S6), while the negative regulators of CK were significantly downregulated in the *zat18* mutants (Figure 7A). Based on these results, we speculated that ZAT18 regulated the expression of CK signaling mainly through modulating the expression of these repressors.

To further confirm the regulation of ZAT18 on the signaling of these hormones, we checked the expression levels of these hormone-related DEGs in two *ZAT18-OE* lines and observed opposite expression patterns from them compared with the *zat18* mutants (Figure 7B). These results suggested that ZAT18 promoted the plant defense and inhibited plant growth under biotic and abiotic stress conditions by differently regulating the expression of defense-related hormones (SA, ABA and ET) and growth-related hormones (auxin and CK).

## 3. Discussion

**Figure 8:**
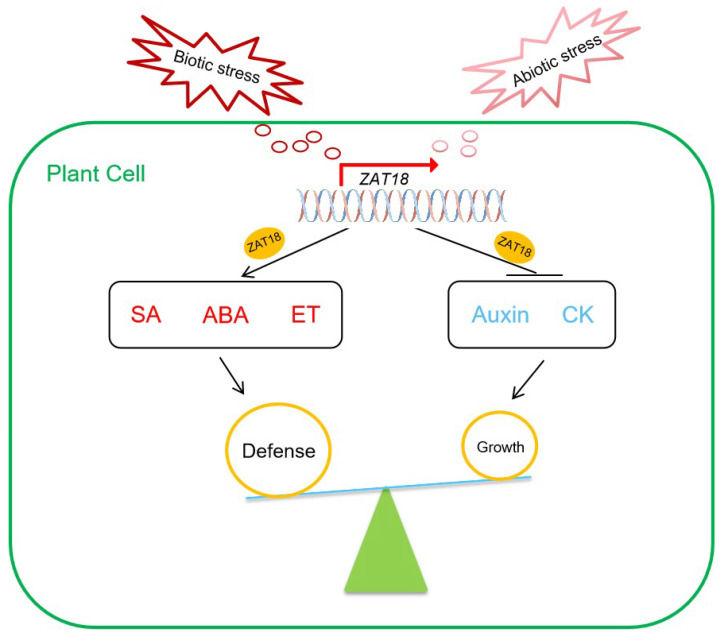
The working model of ZAT18. Under stress conditions, the expression of *ZAT18* is induced. As a transcription factor, ZAT18 induces plant defense by upregulating the SA, ET and ABA pathways. Meanwhile, the auxin and CK pathways are repressed by ZAT18 to inhibit plant growth.

Activation of defense responses under unfavorable conditions is usually accompanied by growth inhibition, probably because of the competition for resources [3,10]. To balance growth and defense, plants use multiple strategies, including hormone crosstalk. Defense-related hormones are synthesized under stress conditions, and their signaling is activated to defend against environmental stresses [39,40,41,42]. Meanwhile, they also negatively regulate pathways of growth-related hormones to inhibit growth [19,43,44]. Compared with enzymes that function in protein modification, transcription factors that directly change the transcriptome have been less explored in the crosstalk of growth and defense hormones. In this study, we found that ZAT18 was upregulated by the defense-related hormone SA and pathogens to promote SA signaling for pathogen resistance. In addition, ZAT18 inhibited auxin signaling, indicating that ZAT18 was a transcription factor regulating the growth-defense tradeoff upon pathogen infection by modulating the crosstalk of SA and auxin. Further transcriptome analysis suggested that ZAT18 was also a positive regulator of the ABA and ET pathways and a negative regulator of CK signaling. Combined with ZAT18 being activated by multiple abiotic stresses and enhancing plant defense to drought in a previous report [31], we proposed that ZAT18 was upregulated by both abiotic and biotic stresses to balance growth and defense by fine-tuning the antagonistic interactions between the signaling of growth-related hormones (auxin and CK) and defense-related hormones (SA, ET and ABA) at the transcriptional level (Figure 8).

Among the zinc finger proteins, the ZAT family is the better-characterized subclass in defense responses. Even though most studies on ZATs are on the controls of abiotic stresses [24,26,30,45], the connections of ZATs with biotic stresses were also reported. Shi et al. found that ZAT6 is induced by pathogen infection and enhances plant immunity by modulating SA synthesis and signaling [46]. ZAT6 is also a target of mitogen-activated protein kinase 6 (MPK6), which can be activated by pathogen infection, indicating that plants may activate ZAT6-mediated defense responses at both the transcriptional and posttranslational levels [47,48]. During the preparation of this manuscript, ZAT18 was reported to be a regulator of immunity by modulating the crosstalk of SA and pathogen-secreted coronatine [49]. In this Gao et al. report, ZAT18 enhanced susceptibility instead of resistance to pathogens and suppressed SA signaling during pathogen infection through coronatine [49]. We hypothesize that these inconsistencies may have been due to different pathogen strains (*Psm* ES4326 vs. *Pst* DC3000) or different infection methods (spray inoculation vs. infiltration) used in the two studies. Under their conditions, the antagonistic function of coronatine on SA may be stronger, which concealed the promotion of SA signaling by ZAT18.

Our qRT-PCR results showed that SA could induce the expression of *ZAT18* in the *npr1-2* mutant (Figure 1B), indicating that ZAT18-mediated auxin repression was NPR1-independent. This is consistent with previous findings showing that SA suppresses plant growth that relies on SA-binding proteins (SABPs) other than NPRs [20,50,51]. Thus, other SABPs may bind SA to induce the expression of ZAT18 after SA treatment [52]. Moreover, the expression of *ZAT18* can be upregulated by many elicitors, such as pathogens, salt, drought and heat [31], and ZAT18 promotes plant defense against biotic and abiotic stresses, indicating that *ZAT18* is a general stress-responsive gene for defense activation and growth inhibition. It will be interesting to investigate whether stresses elevate the transcription of *ZAT18* through different mechanisms or whether a common regulator exists to combine stress signaling for ZAT18 activation.

In summary, we identified a C2H2-type transcription factor, namely, ZAT18, which oppositely regulated SA and auxin signaling to promote defense and inhibit growth. Moreover, our transcriptome data suggested that ZAT18 also regulated the signaling of other defense- and growth-related hormones. It is interesting that different expression levels and/or tag-fusing forms (GFP-ZAT18 or ZAT18-GFP) of *ZAT18-OE* lines change the growth phenotype but not defense responses [31,49]. Thus, further exploration of the mechanisms by which ZAT18 differentially regulates growth and defense may help us to find a way to enhance defense without growth penalties for crop molecular breeding.

## 4. Materials and Methods

### 4.1. Plant Materials, Growth Conditions and Phytohormone Treatments

The *Arabidopsis thaliana* wild type (WT), mutants and transgenic plants used in this study were all from the Colombia-0 ecotype background. The *zat18-1* (SALK_027144C) was an Arabidopsis T-DNA insertion mutant and was obtained from the Arabidopsis Biological Resource Center (ABRC). Homozygotes were identified via PCR using the primers ZAT18TM-LP, ZAT18TM-RP and LBb1.3, which are listed in Table S2. The *npr1-2* and *tga256* mutants were described previously [53,54]. The *zat18cr* and *ZAT18-OE #9*/*#12* were transgenic plants created in our lab.

Plants grown in soil were cultivated at 22 °C in a greenhouse with a 12 h light/12 h dark cycle. For plant seedling cultivation, seeds were sterilized with 75% ethanol for 1 min, rinsed once with sterile dH_2_O and then sterilized with 1% sodium hypochlorite for 8–10 min before being rinsed three times with sterile dH_2_O. Sterile seeds were vernalized at 4 °C for 3 days. After that, plants were grown on solid 1/2 MS medium with 15 g/L sucrose and were maintained at 22 °C with a 16 h light/8 h dark cycle.

For the benzothiadiazole (BTH) treatment in the pathogen infiltration assays, three-week-old plants were sprayed with 120 µM BTH and were maintained in the dark for 24 h under normal growth conditions. For the total RNA extraction assay, two-week-old seedlings were dipped in 1 mM SA solution or control solution for a specified time before collection for RNA extraction.

### 4.2. Pathogen Inoculation

In this study, *Pseudomonas syringae* pv.*maculicola* (*Psm*) ES4326 was used for the pathogen inoculation. *Psm* ES4326 was grown at 28 °C in King’s B medium containing 50 mg/L streptomycin for approximately 36 h. Bacterial cultures were collected and suspended with 10 mM MgSO_4_ and then diluted to a final optical density of 0.01 or 0.1 at 600 nm (OD_600nm_). The diluted bacterial suspension with 0.02% Silwet L-77 was sprayed on the leaves of three-week-old Arabidopsis. Three days later, the inoculated third to sixth true leaves were weighed and collected for grounding in 10 mM MgSO_4_ and then diluted to five concentrations in a tenfold decreasing gradient for the bacterial biomass detection assay.

### 4.3. Construction for Creating Transgenic Plants

For the CRISPR/Cas9 mediated genome editing in plants, a 20 nt specific spacer against the exon of *ZAT18* with the AGG protospacer-adjacent motif (PAM) sequence (ZAT18gRNA-R) and its reverse complementary sequence (ZAT18gRNA-F) were artificially synthesized (Table S2). The annealed products of these two primers were ligated into vector pHEE401E [55] and transformed into competent cells of *E. coli* strain DH5α. The correct construct (pHEE401E::ZAT18gRNA) was then transformed into *Agrobacterium* strain GV3101 using a heat shock transition.

For creating the *ZAT18* overexpression plants, the full-length coding sequence of *ZAT18* was ligated into vector pART27 with a GFP tag [56]. The correct construct pART27::ZAT18-GFP was transformed into *Agrobacterium* strain GV3101 using a heat shock transition for further experiments. The primers used in this experiment are listed in Table S2.

The *Arabidopsis thaliana* ecotype Colombia-0 was used for transformation with *Agrobacterium tumefaciens* carrying correct constructs (pHEE401E::ZAT18gRNA or pART27::ZAT18-GFP), as described previously [56,57]. Briefly, the transformed *Agrobacterium tumefaciens* were incubated overnight in Luria-Bertani medium with 50 mg/L kanamycin and 50 mg/L rifampicin for pHEE401E::ZAT18gRNA, or 100 mg/L spectinomycin and 50 mg/L rifampicin for pART27::ZAT18-GFP, at 28 °C and 200 rpm. Then, the bacteria were pelleted and resuspended in an infiltration buffer (10 mM 2-(N-morpholino) ethane sulphonic acid (MES), 10 mM MgCl_2_ and 200 mM acetosyringone, pH 5.6). The flowering Arabidopsis was dipped into a suspension mixture for 10 to 30 s and was cultivated overnight in the dark before growth under normal conditions. The positive transformants of *ZAT18* overexpression in T1 generation were screened on solid 1/2 MS medium with 50 mg/L kanamycin. Then, the T1 plants with a single T-DNA insertion were used to obtain *ZAT18-OE* homozygous lines (*#9* and *#12*) in the T3 generation. Plants carrying the pHEE401E::ZAT18gRNA construct were screened on solid 1/2 MS medium with 25 mg/L hygromycin. The homozygous genome-edited plant was identified via Sanger sequencing using primer ZAT18-F.

### 4.4. Total RNA Extraction and qRT-PCR

Total RNA was extracted using a TRIzol reagent. For testing the expression pattern of *ZAT18* induced by SA or the pathogen, two-week-old seedlings or leaves of three-week-old plants were collected at 0, 1, 3, 6, 12 and 24 h post-inoculation (hpi) respectively. For testing the expression levels of *WRKY54* and *WRKY70* after SA treatment, two-week-old seedlings were collected at 1 hpi, as *WRKY54* and *WRKY70* are SA early responsive genes. For testing the expression levels of marker genes in different hormone signaling pathways in the *ZAT18-OE* lines and *zat18* mutants, leaves of three-week-old plants without any treatment were collected. First-strand complementary DNA was synthesized from total RNA using a reverse transcription kit [HiScript^®^ III RT SuperMix for qPCR (+gDNA wiper), R323-01, Vazyme Biotech Co., Ltd, Nanjing, China]. qRT-PCR was performed using a ChamQ SYBR qPCR Master Mix (Q311, Vazyme Biotech Co., Ltd, Nanjing, China) on a QuantStudio 5 Real-Time PCR System (Applied Biosystems QuantStudio 5, Thermo Fisher Scientific, Waltham, MA, USA). *UBQ5* was used as an internal control and the primers UBQ5-qF/qR are listed in Table S2. The expression level of *ZAT18* was determined using the primers ZAT18-qF and ZAT18-qR listed in Table S2. All primers for qRT-PCR are listed in Table S2.

### 4.5. Transcriptome Sequencing

Leaves of three-week-old Arabidopsis, both WT (Colombia-0) and *zat18-1*, were collected as three biological replicates for RNA extraction and RNA-seq. Transcriptome sequencing was performed using the Illumina NovaSeq6000 platform from Biomarker Bioinformatics Technology Co., Ltd. (Qingdao, China). The clean reads were mapped to the Arabidopsis genome (Tair11). Gene expression levels were expressed as fragments per kilobase of exon model per million reads mapped (FPKM). The DEGSeq R package (*p* value < 0.01 and Fold Change ≥ 1.5) was used for differential expression analysis. Gene Ontology (GO) term enrichments were analyzed with the BMKCloud platform (www.biocloud.net, accessed on 2 January 2021).

### 4.6. Statistical Analyses

Group comparisons in the SA or BTH induction experiments were calculated using two-way ANOVA analysis (experimental design: no matching, use regular two-way ANOVA, no repeated measures) in GraphPad Prism 7. The significant differences of all other experiments were determined using Student’s *t* test (experimental design: non parametric tests, Mann-Whitney test, compare ranks) in GraphPad Prism 7. The sample size n can be found in the figure legends, where n means the number of biological replicates unless specifically stated. All data are shown as mean ± s.d. * *p* < 0.05; ** *p* < 0.01; *** and **** *p* < 0.001; ns, no significant difference.

### 4.7. The Measurements of Root Length, Fresh Weight, Cell Size and Cell Number

For measuring the fresh weight of seedlings, 100 ten-day-old seedlings were collected as 10 groups and each group was quantified by scale. For checking the root length, the primary root of each two-week-old seedling was measured from the stem base to the tip using Image J.

For the cell size measurement, the fully expanded fifth and sixth true leaves of three-week-old plants were fixed and decolorized in the clearing solution (75% (*v*/*v*) ethanol and 25% (*v*/*v*) acetic acid). Next, the leaves were cleaned with different concentrations of ethanol solutions (60%, 40%, 20% and 10%) were applied in order. After that, the solution was replaced with the chloral hydrate solution (250 g chloral hydrate dissolved in 100 mL dH_2_O). Mesophyll cells were examined and photographed under a 40× objective lens and the cell size was measured using Image J. For counting the cell number per leaf, the leaf area was measured using Image J and the cell number was determined using the leaf area/cell size.

## Data Availability

All data generated by this study is available upon request.

## Supplementary Materials

The following supporting information can be downloaded from https://www.mdpi.com/article/10.3390/ijms232315436/s1. Figure S1: The relative expression levels of *ZAT18* in the RNA-sequencing data. Figure S2: Identification of the T-DNA insertion mutant of *ZAT18*. Figure S3: The expression levels of *PR* genes in the *ZAT18-OE* lines. Figure S4: Abolishing the function of *ZAT18* had no obvious effects on plant growth. Figure S5: Auxin signaling was activated in the *zat18* mutants with no obvious phenotype. Figure S6: The expression levels of cytokinin-activated genes in the *zat18* mutants. Table S1: Sequencing metrics of the 6 RNA-seq libraries. Table S2: The primers used in this study.

## References

[1] Ngou B.P.M., Ding P.T., Jones J.D.G. (2022). Thirty years of resistance: Zig-zag through the plant immune system. Plant Cell.

[2] Jones J.D., Dangl J.L. (2006). The plant immune system. Nature.

[3] Huot B., Yao J., Montgomery B.L., He S.Y. (2014). Growth-defense tradeoffs in plants: A balancing act to optimize fitness. Mol. Plant.

[4] Hammoudi V., Fokkens L., Beerens B., Vlachakis G., Chatterjee S., Arroyo-Mateos M., Wackers P.F.K., Jonker M.J., van den Burg H.A. (2018). The Arabidopsis SUMO E3 ligase SIZ1 mediates the temperature dependent trade-off between plant immunity and growth. PLoS Genet..

[5] Liu X., Yin Z., Wang Y., Cao S., Yao W., Liu J., Lu X., Wang F., Zhang G., Xiao Y. (2022). Rice cellulose synthase-like protein OsCSLD4 coordinates the trade-off between plant growth and defense. Front. Plant Sci..

[6] Liu N., Xu Y., Li Q., Cao Y., Yang D., Liu S., Wang X., Mi Y., Liu Y., Ding C. (2022). A lncRNA fine-tunes salicylic acid biosynthesis to balance plant immunity and growth. Cell Host Microbe.

[7] Lee H., Khatri A., Plotnikov J.M., Zhang X.C., Sheen J. (2012). Complexity in differential peptide-receptor signaling: Response to Segonzac et al. and Mueller et al. commentaries. Plant Cell.

[8] Pajerowska-Mukhtar K.M., Wang W., Tada Y., Oka N., Tucker C.L., Fonseca J.P., Dong X. (2012). The HSF-like transcription factor TBF1 is a major molecular switch for plant growth-to-defense transition. Curr. Biol..

[9] Xu G., Yuan M., Ai C., Liu L., Zhuang E., Karapetyan S., Wang S., Dong X. (2017). uORF-mediated translation allows engineered plant disease resistance without fitness costs. Nature.

[10] Van Butselaar T., Van den Ackerveken G. (2020). Salicylic Acid Steers the Growth-Immunity Tradeoff. Trends Plant Sci..

[11] Berens M.L., Wolinska K.W., Spaepen S., Ziegler J., Nobori T., Nair A., Kruler V., Winkelmuller T.M., Wang Y., Mine A. (2019). Balancing trade-offs between biotic and abiotic stress responses through leaf age-dependent variation in stress hormone cross-talk. Proc. Natl. Acad. Sci. USA.

[12] Campos M.L., Yoshida Y., Major I.T., de Oliveira Ferreira D., Weraduwage S.M., Froehlich J.E., Johnson B.F., Kramer D.M., Jander G., Sharkey T.D. (2016). Rewiring of jasmonate and phytochrome B signalling uncouples plant growth-defense tradeoffs. Nat. Commun..

[13] Peng Y., Yang J., Li X., Zhang Y. (2021). Salicylic Acid: Biosynthesis and Signaling. Annu. Rev. Plant Biol..

[14] Rekhter D., Ludke D., Ding Y., Feussner K., Zienkiewicz K., Lipka V., Wiermer M., Zhang Y., Feussner I. (2019). Isochorismate-derived biosynthesis of the plant stress hormone salicylic acid. Science.

[15] Torrens-Spence M.P., Bobokalonova A., Carballo V., Glinkerman C.M., Pluskal T., Shen A., Weng J.K. (2019). PBS3 and EPS1 Complete Salicylic Acid Biosynthesis from Isochorismate in *Arabidopsis*. Mol. Plant.

[16] Liu Y., Sun T., Sun Y., Zhang Y., Radojicic A., Ding Y., Tian H., Huang X., Lan J., Chen S. (2020). Diverse Roles of the Salicylic Acid Receptors NPR1 and NPR3/NPR4 in Plant Immunity. Plant Cell.

[17] Kumar S., Zavaliev R., Wu Q., Zhou Y., Cheng J., Dillard L., Powers J., Withers J., Zhao J., Guan Z. (2022). Structural basis of NPR1 in activating plant immunity. Nature.

[18] Pokotylo I., Hodges M., Kravets V., Ruelland E. (2021). A ménage à trois: Salicylic acid, growth inhibition, and immunity. Trends Plant Sci..

[19] Li A., Sun X., Liu L. (2022). Action of Salicylic Acid on Plant Growth. Front. Plant Sci..

[20] Tan S., Abas M., Verstraeten I., Glanc M., Molnar G., Hajny J., Lasak P., Petrik I., Russinova E., Petrasek J. (2020). Salicylic Acid Targets Protein Phosphatase 2A to Attenuate Growth in Plants. Curr. Biol..

[21] Yu X., Cui X., Wu C., Shi S., Yan S. (2022). Salicylic acid inhibits gibberellin signaling through receptor interactions. Mol. Plant.

[22] Wang D., Pajerowska-Mukhtar K., Culler A.H., Dong X. (2007). Salicylic acid inhibits pathogen growth in plants through repression of the auxin signaling pathway. Curr. Biol..

[23] Xie M., Sun J., Gong D., Kong Y. (2019). The Roles of Arabidopsis C1-2i Subclass of C2H2-type Zinc-Finger Transcription Factors. Genes.

[24] Mittler R., Kim Y., Song L., Coutu J., Coutu A., Ciftci-Yilmaz S., Lee H., Stevenson B., Zhu J.K. (2006). Gain- and loss-of-function mutations in Zat10 enhance the tolerance of plants to abiotic stress. FEBS Lett..

[25] Sakamoto H., Maruyama K., Sakuma Y., Meshi T., Iwabuchi M., Shinozaki K., Yamaguchi-Shinozaki K. (2004). Arabidopsis Cys2/His2-type zinc-finger proteins function as transcription repressors under drought, cold, and high-salinity stress conditions. Plant Physiol..

[26] Rossel J.B., Wilson P.B., Hussain D., Woo N.S., Gordon M.J., Mewett O.P., Howell K.A., Whelan J., Kazan K., Pogson B.J. (2007). Systemic and intracellular responses to photooxidative stress in *Arabidopsis*. Plant Cell.

[27] Sakamoto H., Araki T., Meshi T., Iwabuchi M. (2000). Expression of a subset of the *Arabidopsis* Cys(2)/His(2)-type zinc-finger protein gene family under water stress. Gene.

[28] Dang F., Li Y., Wang Y., Lin J., Du S., Liao X. (2022). *ZAT10* plays dual roles in cadmium uptake and detoxification in *Arabidopsis*. Front. Plant Sci..

[29] Nguyen X.C., Kim S.H., Lee K., Kim K.E., Liu X.M., Han H.J., Hoang M.H., Lee S.W., Hong J.C., Moon Y.H. (2012). Identification of a C2H2-type zinc finger transcription factor (ZAT10) from *Arabidopsis* as a substrate of MAP kinase. Plant Cell Rep..

[30] Ciftci-Yilmaz S., Morsy M.R., Song L., Coutu A., Krizek B.A., Lewis M.W., Warren D., Cushman J., Connolly E.L., Mittler R. (2007). The EAR-motif of the Cys2/His2-type zinc finger protein Zat7 plays a key role in the defense response of *Arabidopsis* to salinity stress. J. Biol. Chem..

[31] Yin M., Wang Y., Zhang L., Li J., Quan W., Yang L., Wang Q., Chan Z. (2017). The Arabidopsis Cys2/His2 zinc finger transcription factor ZAT18 is a positive regulator of plant tolerance to drought stress. J. Exp. Bot..

[32] Ding Y., Sun T., Ao K., Peng Y., Zhang Y., Li X., Zhang Y. (2018). Opposite Roles of Salicylic Acid Receptors NPR1 and NPR3/NPR4 in Transcriptional Regulation of Plant Immunity. Cell.

[33] Wang D., Amornsiripanitch N., Dong X. (2006). A genomic approach to identify regulatory nodes in the transcriptional network of systemic acquired resistance in plants. PLoS Pathog..

[34] Kong X., Pan W., Sun N., Zhang T., Liu L., Zhang H. (2021). *GLABRA2*-based selection efficiently enriches Cas9-generated nonchimeric mutants in the T1 generation. Plant Physiol..

[35] Zhang Y., Li X. (2019). Salicylic acid: Biosynthesis, perception, and contributions to plant immunity. Curr. Opin. Plant Biol..

[36] Okumura K., Goh T., Toyokura K., Kasahara H., Takebayashi Y., Mimura T., Kamiya Y., Fukaki H. (2013). GNOM/FEWER ROOTS is Required for the Establishment of an Auxin Response Maximum for Arabidopsis Lateral Root Initiation. Plant Cell Physiol..

[37] Goh T., Kasahara H., Mimura T., Kamiya Y., Fukaki H. (2012). Multiple AUX/IAA-ARF modules regulate lateral root formation: The role of *Arabidopsis* SHY2/IAA3-mediated auxin signalling. Philos. Trans. R Soc. Lond. B Biol. Sci..

[38] Chen Q., Dai X., De-Paoli H., Cheng Y., Takebayashi Y., Kasahara H., Kamiya Y., Zhao Y. (2014). Auxin overproduction in shoots cannot rescue auxin deficiencies in Arabidopsis roots. Plant Cell Physiol..

[39] Hou S., Tsuda K. (2022). Salicylic acid and jasmonic acid crosstalk in plant immunity. Essays Biochem..

[40] Muller M. (2021). Foes or Friends: ABA and Ethylene Interaction under Abiotic Stress. Plants.

[41] Lee S.C., Luan S. (2012). ABA signal transduction at the crossroad of biotic and abiotic stress responses. Plant Cell Environ..

[42] Ali M.S., Baek K.H. (2020). Jasmonic Acid Signaling Pathway in Response to Abiotic Stresses in Plants. Int. J. Mol. Sci..

[43] Huang H., Liu B., Liu L., Song S. (2017). Jasmonate action in plant growth and development. J. Exp. Bot..

[44] Xie Q., Essemine J., Pang X., Chen H., Jin J., Cai W. (2021). Abscisic Acid Regulates the Root Growth Trajectory by Reducing Auxin Transporter *PIN2* Protein Levels in *Arabidopsis thaliana*. Front. Plant Sci..

[45] Rehman A., Wang N., Peng Z., He S., Zhao Z., Gao Q., Wang Z., Li H., Du X. (2021). Identification of C2H2 subfamily ZAT genes in *Gossypium* species reveals *GhZAT34* and *GhZAT79* enhanced salt tolerance in Arabidopsis and cotton. Int. J. Biol. Macromol..

[46] Shi H., Wang X., Ye T., Chen F., Deng J., Yang P., Zhang Y., Chan Z. (2014). The Cysteine2/Histidine2-Type Transcription Factor *Zinc Finger of Arabidopsis thaliana6* Modulates Biotic and Abiotic Stress Responses by Activating Salicylic Acid-Related Genes and C-repeat-binding factor genes in *Arabidopsis*. Plant Physiol..

[47] Chen J., Yang L., Yan X., Liu Y., Wang R., Fan T., Ren Y., Tang X., Xiao F., Liu Y. (2016). Zinc-Finger Transcription Factor ZAT6 Positively Regulates Cadmium Tolerance through the Glutathione-Dependent Pathway in Arabidopsis. Plant Physiol..

[48] Chang M., Chen H., Liu F., Fu Z.Q. (2022). PTI and ETI: Convergent pathways with diverse elicitors. Trends Plant Sci..

[49] Gao Y., Li Z., Yang C., Li G., Zeng H., Li Z., Zhang Y., Yang X. (2022). *Pseudomonas syringae* activates *ZAT18* to inhibit salicylic acid accumulation by repressing *EDS1* transcription for bacterial infection. New Phytol..

[50] Rong D., Luo N., Mollet J.C., Liu X., Yang Z. (2016). Salicylic Acid Regulates Pollen Tip Growth through an NPR3/NPR4-Independent Pathway. Mol. Plant.

[51] Yuan H.M., Liu W.C., Lu Y.T. (2017). CATALASE2 Coordinates SA-Mediated Repression of Both Auxin Accumulation and JA Biosynthesis in Plant Defenses. Cell Host Microbe.

[52] Manohar M., Tian M., Moreau M., Park S.W., Choi H.W., Fei Z., Friso G., Asif M., Manosalva P., von Dahl C.C. (2014). Identification of multiple salicylic acid-binding proteins using two high throughput screens. Front. Plant Sci..

[53] Spoel S.H., Mou Z., Tada Y., Spivey N.W., Genschik P., Dong X. (2009). Proteasome-mediated turnover of the transcription coactivator NPR1 plays dual roles in regulating plant immunity. Cell.

[54] Zhang Y.L., Tessaro M.J., Lassner M., Li X. (2003). Knockout analysis of Arabidopsis transcription factors *TGA2*, *TGA5*, and *TGA6* reveals their redundant and essential roles in systemic acquired resistance. Plant Cell.

[55] Xing H.L., Dong L., Wang Z.P., Zhang H.Y., Han C.Y., Liu B., Wang X.C., Chen Q.J. (2014). A CRISPR/Cas9 toolkit for multiplex genome editing in plants. BMC Plant Biol..

[56] Li W.W., Zhao D., Dong J.W., Kong X.L., Zhang Q., Li T.T., Meng Y.L., Shan W.X. (2020). *AtRTP5* negatively regulates plant resistance to *Phytophthora* pathogens by modulating the biosynthesis of endogenous jasmonic acid and salicylic acid. Mol. Plant Pathol..

[57] Clough S.J., Bent A.F. (1998). Floral dip: A simplified method for *Agrobacterium*-mediated transformation of *Arabidopsis thaliana*. Plant J..

