# Supplementary material for: Arabidopsis Cys2/His2 Zinc Finger Transcription Factor ZAT18 Modulates the Plant Growth-Defense Tradeoff
Source: Int J Mol Sci. 2022 Dec 6;23(23):15436. doi: 10.3390/ijms232315436 (PMC9738932; doi:10.3390/ijms232315436)
Supplement: Supplementary file 1 [file ijms-23-15436-s001.zip › ijms-2060935-supplementary.pdf]

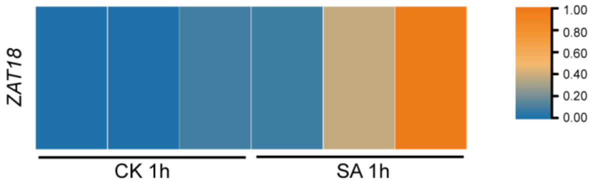

Figure S1. The relative expression levels of *ZAT18* in RNA-sequencing data. Two-week-old WT seedlings were treated with sterile dH_2O (CK) or 1 mM SA for 1 hour, and samples were collected for RNA extraction and sequencing. The expression levels of *ZAT18* in different samples based on FPKM are shown (blue bar indicates lower expression level while orange bar indicates higher expression level). FPKM: fragments per kilobase of exon model per million reads mapped. Each vertical section represents an individual sample and there are three biological repetitions for both CK and SA treatment.

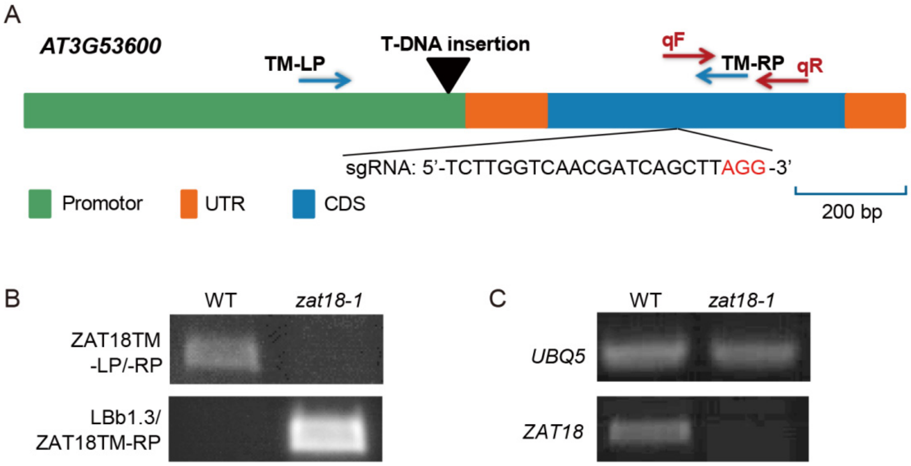

Figure S2. Identification of the T-DNA insertion mutant of *ZAT18*. (**A**) Gene structure of *ZAT18* is represented by promoter (green bar), untranslated regions (orange bars) and coding sequence (blue bar). The T-DNA insertion site and locations of primers for genotyping and qRT–PCR are indicated. The sgRNA target site for creating *zat18cr* is also indicated, and the PAM sequence is highlighted in red. (**B**) DNA was extracted from three-week-old WT and *zat18-1* mutant plants. PCR was performed with the ZAT18TM-LP/-RP and LBb1.3/ZAT18TM-RP sets of primers. (**C**) RNA was extracted from two-week-old WT and *zat18-1* mutant seedlings, and qRT–PCR was performed with *ZAT18*-specific primers (ZAT18-qF/qR) using *UBQ5* as loading controls.

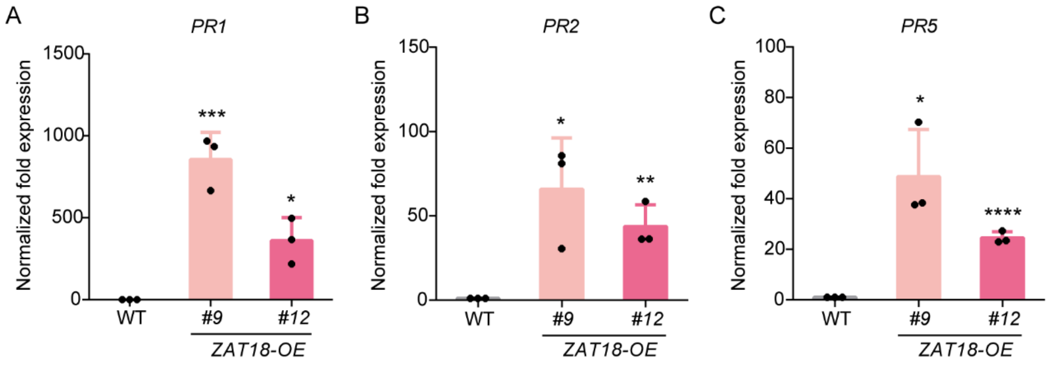

Figure S3. The expression levels of *PR* genes in *ZAT18-OE* lines. Two-week-old WT and *ZAT18-OE* seedlings without any treatment were collected for RNA extraction and qRT–PCR on *PR1* (**A**), *PR2* (**B**) and *PR5* (**C**). The expression levels of *UBQ5* served as references. Significant differences were detected by Student's *t* test. Data are shown as mean ± s.d. (n = 3, n indicates biological replicates). * $p < 0.05$; ** $p < 0.01$; *** $p < 0.001$; **** $p < 0.0001$.

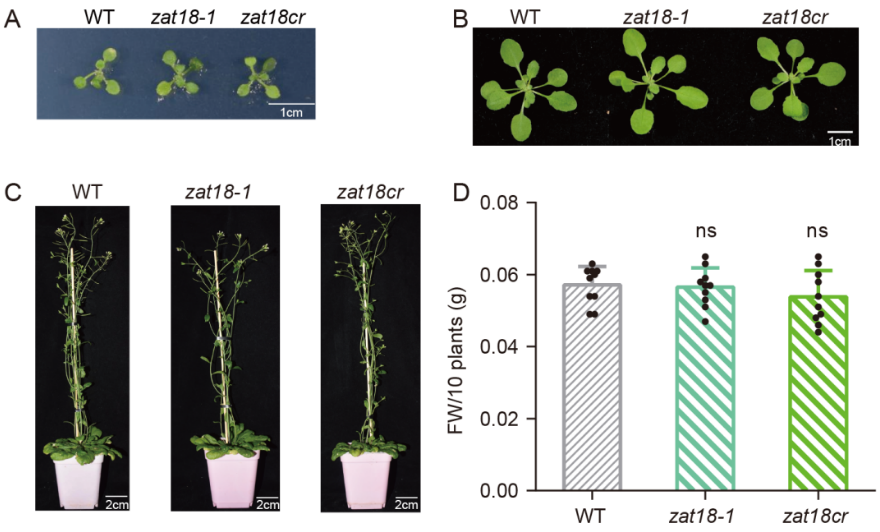

Figure S4. Abolishing the function of *ZAT18* has no obvious effects on plant growth. Representative plants of ten-day-old (**A**), three-week-old (**B**) and eight-week-old (**C**) WT and *zat18* mutants. (**D**) Seedlings of ten-day-old WT and *zat18* mutants were collected and weighed. Data are shown as mean ± s.d. (n = 10, n indicates biological replicates). ns, no significant difference.

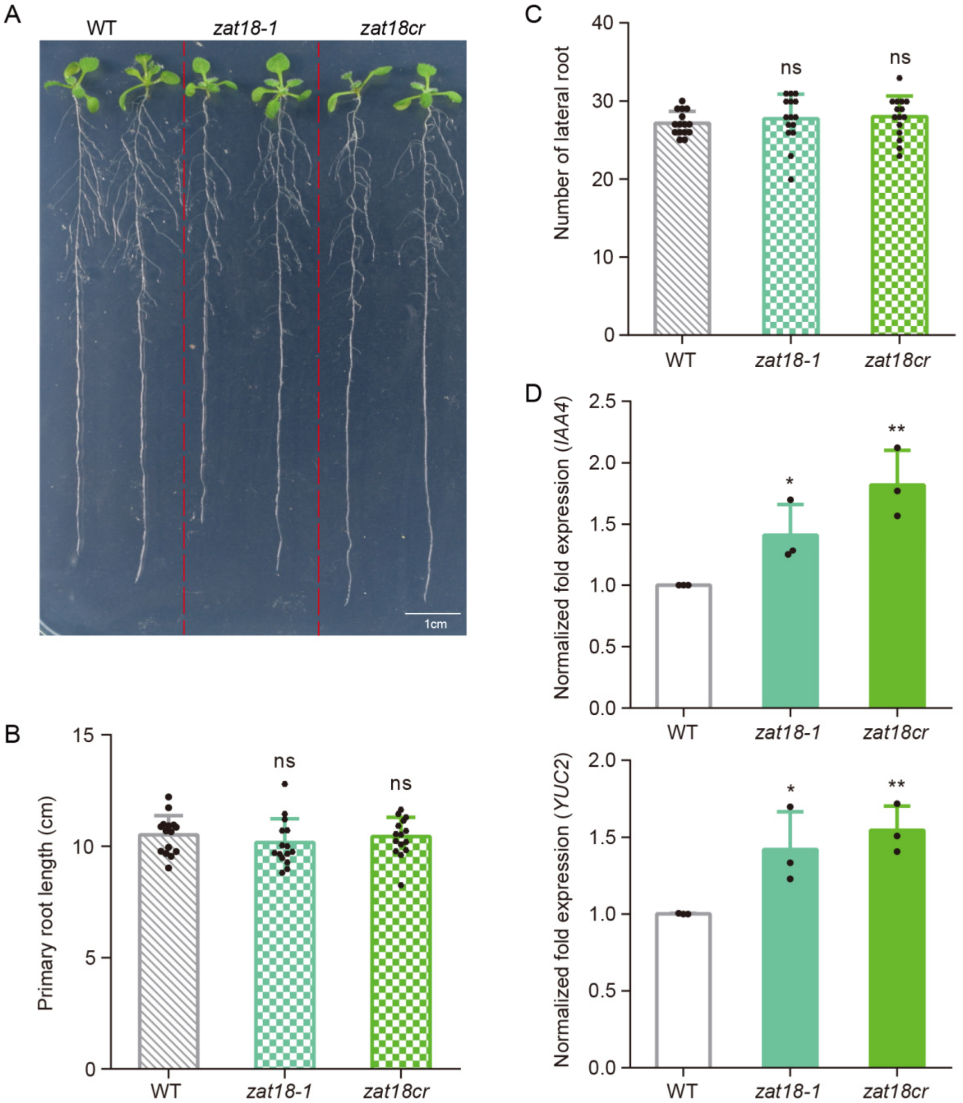

Figure S5. Auxin signaling is activated in *zat18* mutants with no obvious phenotype. (**A**) Representative seedlings to show root growth status of two-week-old WT and *zat18* mutant. (**B**) The primary root lengths of two-week-old WT and *zat18* mutants measured by ImageJ. Data are shown as the mean ± s.d. (n = 15, n indicates biological replicates). (**C**) The numbers of visible lateral roots of two-week-old WT and *zat18* mutants. Data are shown as mean ± s.d. (n = 15, n indicates biological replicates). (**D**) The expression levels of *IAA4* and *YUC2* in two-week-old WT and *zat18* mutant seedlings without any treatment. Data are shown as mean ± s.d. (n = 3, n indicates biological replicates). Significant differences were detected by Student's *t* test. * $p < 0.05$; ** $p < 0.01$; ns, no significant difference.

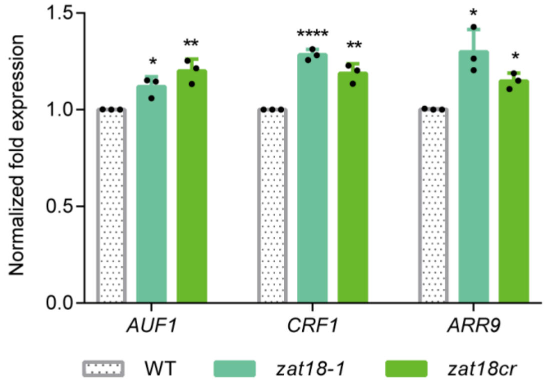

Figure S6. The expression levels of cytokinin activated genes in *zat18* mutants. Two-week-old WT and *zat18* mutant seedlings without any treatment were collected for RNA extraction and qRT–PCR. The expression levels of *UBQ5* served as references. Significant differences were detected by Student's *t* test. Data are shown as mean ± s.d. (n = 3, n indicates biological replicates). * $p < 0.05$; ** $p < 0.01$; **** $p < 0.0001$.

Table S1. Sequencing metrics of the 6 RNA-seq libraries.

Genotypes	Sample ID	Total reads	Mapped Reads	Q30(%)
WT	WT-1	39,376,864 (100%)	38,166,341 (96.93%)	94.88
	WT-2	41,383,362 (100%)	40,114,824 (96.93%)	94.72
	WT-3	42,523,812 (100%)	41,236,038 (96.97%)	94.65
zat18	*zat18*-1	39,180,910 (100%)	37,939,621 (96.83%)	94.97
	zat18-2	40,895,410 (100%)	39,482,637 (96.55%)	93.49
	zat18-3	39,247,270 (100%)	37,803,206 (96.32%)	93.09

Table S2. The primers used in this study.

Primer Name	Sequences (5'-3')	Description
ZAT18-F	TTTCATTTGGAGAGGACACGCTCGAGA TGAAGAGAGACCGGTCCG	Primers for *ZAT18* coding sequence amplification
ZAT18-R	TCCTCGCCCTTGCTCACCATCTCGAGA TTCACAAACTTCAAATCAATTTGTG	
ZAT18gRNA-F	ATTGTCTTGGTCAACGATCAGCTT	Primers for constructing *ZAT18* genome editing vector
ZAT18gRNA-R	AAACAAGCTGATCGTTGACCAAGA	
ZAT18-qF	TAGGACGAGCATGATAACCGA	qRT-PCR primers for *ZAT18*
ZAT18-qR	CAAATTCTTCCCAAAGATTAACAC	
ZAT18TM-LP	ATCGATTGCTCGGTTATCATG	Primers for identification of homozygous T-DNA insertion mutants
ZAT18TM-RP	TTTAAACGCAGTCACGATTCC	
LBb1.3	ATTTTGCCGATTTCGGAAC	
WRKY54-qF	GCACTGCTCAGAACCATGTCAA	qRT-PCR primers for *WRKY54*, *WRKY70*, *ACL5*, *LAX2*, *TAA1*, *YUC2*, *IAA4*, *AUF1*, *CRF1*, *ARR9*, *KMD1/2/4*, *PAD4*, *SARD1*, *RD26*, *PCC1*, *CPK22*, *RAV2*, *ERF018*, *MYB3R-5* and *UBQ5* of Arabidopsis.
WRKY54-qR	CAAGTCCTCACCTGTCGAAGA	
WRKY70-qF	CAAGGCAACAAAGCAAGTCC	
WRKY70-qR	CTTCTTCCTTCATTGAGGTAGATAAAC	
ACL5-qF	GGCTGAATTAGAGAAAAGGGAAGA	
ACL5-qR	GGTGACAAAAATGCCATTAGGG	
LAX2-qF	TCCCAAAGAATCTTTACCGTGA	
LAX2-qR	CTTCTCCCACACAAAGTAGAGT	
TAA1-qF	CCCACTACACTCCCATCAC	
TAA1-qR	TCCTTGTCCTTCACCAATGCC	
YUC2-qF	CAAGATCAAATGCGGAAAGACT	
YUC2-qR	CCGAATAATGCATTACCCGTTT	
IAA4-qF	GGCTCAGACTTTGTACCTACTT	
IAA4-qR	ATCCTTAGCCTCTTACAAGACG	
AUF1-qF	TCTGACGAAATCCTAACCCGA	
AUF1-qR	CCGAACTCAGCCTTCCACTT	
CRF1-qF	TGGAGTGAGGCAAAGACCG	
CRF1-qR	GCGTTATCGTAAACAATGGCAG	
ARR9-qF	GTATGGCAGCAGAATCGCAG	
ARR9-qR	CTTAGAAAACGCATTTGGGTCG	
KMD1-qF	GATGTGATGATGTTTCTAAATGAC	
KMD1-qR	TATCCAGCCAACGCCTTCC	
KMD2-qF	GCGTTCCTTCTTTGCCTGC	
KMD2-qR	AAGCCCATCTGTCTTCTGCC	
KMD4-qF	GAGTCTCAGATGACGCAGC	
KMD4-qR	CGCCGATTAACAACACTTTCC	
PAD4-qF	ACCGAGGAACATCAGAGGTAC	
PAD4-qR	AAATTCGCAATGTCGAGTGGC	
SARD1-qF	CCGTAAGTTTAGAATCGGTGCG	

SARD1-qR	TTGATGTGGCGAGAGGAGAGC	
RD26-qF	TGGGTCGTCATCGTCTTCTTC	
RD26-qR	GTAACTCGGTAATCCATTGGTC	
PCC1-qF	GCAGCAGCAGTGGAGACAA	
PCC1-qR	CGCCGCAGCAGAAGATACA	
CPK22-qF	GACAAAAGCGGGTCAATCACT	
CPK22-qR	GTTCCATTCCCATCAACATCAG	
RAV2-qF	TGATTGGAAAGTTCGGTCTGG	
RAV2-qR	GCTTCACGGTGGTCACATTA	
ERF018-qF	TTGGTTAGGCTCTTACGACAC	
ERF018-qR	GGAGGCGTCAACGACTTTTC	
MYB3R-5-qF	TGATTCGCTTACCCAAACCTC	
MYB3R-5-qR	AGCATACTCATTGACACCGATT	
UBQ5-qF	GTAAACGTAGGTGAGTCCA	
UBQ5-qR	GACGCTTCATCTCGTCC	

Supplementary Materials and Methods

1. Genomic DNA extraction

Leaves of three-week-old plants were collected to grind in liquid nitrogen before extracted by cetyltrimethyl ammonium bromide (CTAB) buffer [2% CTAB, 2% polyvinylpyrrolidone (PVP), 100 mM Tris-HCl pH 8.0, 25 mM ethylene diamine tetraacetic Acid (EDTA), 2 M NaCl]. The mixture was incubate at 65℃ for 20 min and then cooled down to room temperature. The phenol/chloroform/isoamylol buffer in a ratio of 25:24:1 was added into the mixture and mixed violently before centrifuged at 12000 rpm for 15 min. Then we transferred supernatant to a clean tube, mixed it with a same volume of isopropanol, kept the mixture at -20℃ for 1 hour and centrifuged it at 12000 rpm for 15 min to collect the DNA precipitate. The DNA precipitate was cleaned with 80% ethanol twice and dissolved in sterile ddH_2O.

2. RNA extraction and qRT-PCR

As description in Materials and Methods section 4.4 of main text.

3. Statistical analyses

As description in Materials and Methods section 4.6 of main text.

4. The measurements of root length, fresh weight, cell size and cell number

As description in Materials and Methods section 4.7 of main text.
